# Assessing the role of initial conditions in the local structural identifiability of large dynamic models

**DOI:** 10.1038/s41598-021-96293-9

**Published:** 2021-08-19

**Authors:** Dominique Joubert, J. D. Stigter, Jaap Molenaar

**Affiliations:** Biometris, Department of Mathematical and Statistical Methods, Wageningen University and Research, 6708 PD Wageningen, The Netherlands

**Keywords:** Environmental sciences, Engineering, Mathematics and computing, Scientific data, Software, Computational biology and bioinformatics, Predictive medicine, Software

## Abstract

*Structural identifiability* is a binary property that determines whether or not *unique* parameter values can, in principle, be estimated from error-free input–output data. The many papers that have been written on this topic collectively stress the importance of this a priori analysis in the model development process. The story however, often ends with a structurally unidentifiable model. This may leave a model developer with no plan of action on how to address this potential issue. We continue this model exploration journey by identifying one of the possible sources of a model’s unidentifiability: problematic initial conditions. It is well-known that certain *initial values* may result in the loss of local structural identifiability. Nevertheless, literature on this topic has been limited to the analysis of small toy models. Here, we present a systematic approach to detect problematic initial conditions of real-world systems biology models, that are usually not small. A model’s identifiability can often be reinstated by changing the value of such problematic initial conditions. This provides modellers an option to resolve the “unidentifiable model” problem. Additionally, a good understanding of which initial values should rather be avoided can be very useful during experimental design. We show how our approach works in practice by applying it to five models. First, two small benchmark models are studied to get the reader acquainted with the method. The first one shows the effect of a zero-valued problematic initial condition. The second one illustrates that the approach also yields correct results in the presence of input signals and that problematic initial conditions need not be zero-values. For the remaining three examples, we set out to identify key initial values which may result in the structural *unidentifiability*. The third and fourth examples involve a systems biology Epo receptor model and a JAK/STAT model, respectively. In the final Pharmacokinetics model, of which its global structural identifiability has only recently been confirmed, we indicate that there are still sets of initial values for which this property does not hold.

## Introduction

We have grown to appreciate the importance of model accuracy as societies increasingly depend on model predictions to answer difficult questions, such as which measures should be taken to fight the Covid-19 pandemic. Clearly, mathematical models are powerful tools. They often contain unknown parameters which need to be estimated from experimental data since they are not measurable. Model developers often run into practical difficulties whilst estimating the values of such parameters. These range from significant computational memory requirements to insufficient funding to perform required experiments^1^. Here we highlight *model identifiability*, one of the primary challenges encountered during in parameter estimation. We focus on the role *initial conditions* play in a model’s local structural identifiability.

Parameter identifiability assesses whether it is possible to infer *unique* parameter values from gathered input-output data. It can be divided into two main categories with structural or a priori identifiability the prerequisite for practical identifiability. Structural identifiability is based on a model’s structure and is a binary property and so supposes that experimental measurements are error free. In contrast, practical identifiability characterises the ability to estimate parameters from observed data containing measurement errors and so takes both data quality and availability into account^2^.

Developing dynamic models using ordinary differential equations requires the formulation of differential (and possibly accompanying algebraic equations) and calls for the definition of measurable outputs and initial conditions. A model’s structural identifiability can be affected by these chosen initial values^3–5^. It may therefore be impossible to uniquely estimate certain parameters if a system that evolves from a “problematic set of initial conditions”. In 2001 Denis-Vidal and co-authors alluded to this by stressing the importance of an appropriate choice of initial values^3^. This was confirmed by Saccomani et al.^4^ who studied the role initial conditions play in the identifiability of controlled models. They commented that “it happens frequently in global identifiability analyses that the property only holds generically, i.e. except for a ‘thin’ set of initial conditions. In these situations the system is (incorrectly but forgivably) declared to be (global) identifiable”^4^. Finally, Villaverde and Banga remarked that both the symbolic differential algebra and differential geometry methods, which are often used to assess a model’s identifiability, do not recognise the loss of local structural identifiability for very specific initial values^5^. Furthermore, they list a number of methods that can detect these problematic initial conditions, including their own differential geometry based approach. These methods include^5^:

- Exact Arithmetic Rank method (EAR)—This method works for rational systems was introduced in 2012 by Karlsson et al.^6^. It allows for the definition of specific initial values and uses these in efficient numerical calculations.
- DAISY—This method also analyses rational systems exclusively and uses the differential algebra method^7^.
- STRIKE-GOLDD—This method adopts a differential geometry approach to analyse a model’s local structural identifiability and so entails the symbolical calculation of successive Lie derivatives^8^.

It should be mentioned that some methods do not always yield correct results for certain special cases. In the “Discussion” we mention such a case explicitly. The method we present in this paper has a higher degree of reliability since it combines 2 analyses, one numerical and one symbolic. It is also not restricted to rational or relatively small models. As extensively described in the “Methods” section, our approach is based on the identifiability algorithm presented by Stigter and Molenaar^9^. The method starts with a numerical analysis of the rank of the so-called sensitivity matrix and is available as a downloadable application^10^. This initial step pinpoints potentially unidentifiable parameters and initial conditions. In the second step a symbolic analysis is performed to check whether these parameters and initial conditions are indeed causing identifiability problems. Since this analysis only involves a restricted number of parameters and initial conditions, the computational demand which is often found to be the curse of a symbolic analysis of the full model, is significantly reduced. The numerical results from the first step can attractively be summarised in a so-called “identifiability signature”^11^. This signature contains a graphical presentation of the singular values resulting from a Singular Value Decomposition (SVD) of the sensitivity matrix. Structural unidentifiability is indicated by a *clear gap in the displayed singular values*. Furthermore, the signature shows the components of the singular vectors corresponding to the close-to-zero singular values. The nonzero components of these vectors reveal which parameters and initial conditions are expected to be unidentifiable.

An advantage of structural identifiability is that it can be assessed before the experimental phase. Since the method presented in this paper allows for the efficient identification of problematic initial conditions, the values of these may be altered before any expenses on wasteful experiments are incurred. So, it may play an essential role in the design-of-experiment stage that should precede any experimental endeavour.

## Results

The discussion of problematic initial conditions has in the past been limited to small toy models. To show the effectiveness and power of the present approach, we apply the method to 5 models. These examples have been carefully chosen to demonstrate different aspects of the approach. First, 2 small benchmark models are studied to get the reader acquainted with the method. The first one shows the effect of a zero-valued problematic initial condition. The second illustrates that the approach also yields correct results in the presence of input signals and that problematic initial values need not be zero-values. The third example comprises a realistic systems biology model describing Epo receptor dynamics. For this Epo model we show that it is possible - thanks to the efficiency of the approach - to identify the precise (zero-valued) initial conditions that result in model unidentifiability. The fourth example is an analysis of the well-known JAK/STAT model, for which the cause of its unidentifiability was not yet published in the literature. In the last example, of which its global structural identifiability has only recently been confirmed, we indicate that there are still sets of initial values for which this property does not hold.

### Example 1

*Small benchmark model* ($M_1$).

In example 1 we analyse a small academic model published by Denis-Vidal et al.^3^. This example illustrates the potential role initial values play in the structural identifiability of uncontrolled models. The model contains two state equations:

1 $$\begin{aligned} \frac{dx_{1}}{dt}&= p_1x_1^2+p_2x_1x_2, \qquad x_1(0) = x_{10} \ne 0, \end{aligned}$$

2 $$\begin{aligned} \frac{dx_{2}}{dt}&= p_3x_1^2+x_1x_2, \qquad x_2(0) = 0. \end{aligned}$$

**Figure 1:**
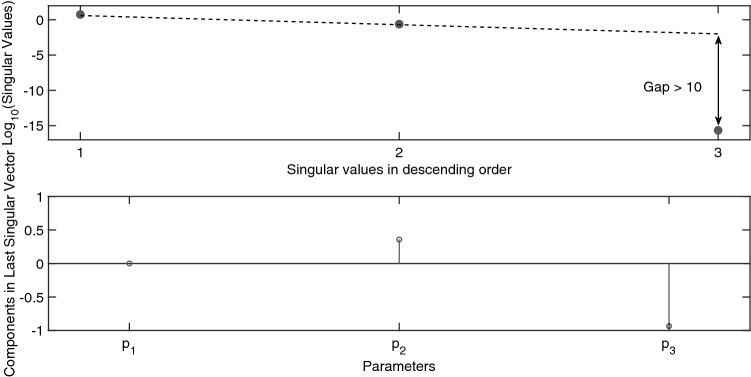
Identifiability signature of Example 1: small benchmark model ($M_1$). (Top) Singular values: For $x_2(0) = 0$ and $y = x_1$, the rank deficiency of the sensitivity matrix is indicated by a distinct gap between the second and third singular values. This suggests that this model is structurally unidentifiable and that there is a single set of totally correlated parameters. (Bottom) Elements of the singular vector related to the zero valued singular value. The nonzero components indicate that parameters $p_2$ and $p_3$ are structurally unidentifiable.

The first state is measured directly, so the output is $y=x_1$. The three system parameters are assumed unknown and therefore the parameter vector is $\varvec{\theta }=[p_1, p_2,$ $p_3]$. In Fig. 1 we present the identifiability signature of this model resulting from the numerical analysis; see “Methods” section for details. It shows a clear gap between the second and third singular values, which indicates that the model is structurally unidentifiable given the set of initial conditions in Eqs. (1) and (2). Our result is in line with the analysis by Denis-Vidal et al. which shows that parameters $p_2$ and $p_3$ are structurally unidentifiable^3^. At the bottom of Fig. 1 the components of the singular vector corresponding to the close-to-zero singular value of the sensitivity matrix are given. The nonzero components correspond to parameters $p_2$ and $p_3$ respectively; this indicates that these parameters are indeed unidentifiable. The second step in our method is to check these numerical suggestions symbolically. The parameter set to be analysed is now reduced to $\varvec{\theta ^{unid}}=\{p_2,p_3 \}$ (obtained from Fig. 1). The Jacobi matrix needed in this second step (see the “Methods” section), is given in Eq. (3) as a $4 \times 2$ matrix, with each column related to a parameter in $\varvec{\theta ^{unid}}$. It is computed by calculating partial derivatives of successive Lie derivatives, defined in Eqs. (56) and (57).

3 $$\begin{aligned} \frac{d {\varvec{G}}}{d \varvec{\theta ^{unid}}}(\varvec{x(0), \theta }) = \begin{pmatrix} 0 &{} 0 \\ 0 &{} 0 \\ p_3 &{} p_2 \\ (1+6p_1)p_3 &{} (1+6p_1)p_2 \\ \end{pmatrix}. \end{aligned}$$

The null-space of the matrix in Eq. (3) is ${\mathcal {N}} \left( \frac{d {\varvec{G}}}{d \varvec{\theta ^{unid}}}(\varvec{x(0), \theta }) \right) =\{-\frac{p_2}{p_3}, 1 \}$. Entries of the base-vector of this nontrivial null-space are the coefficients of the partial differential equation that describes the linear dependence between the 2 columns of this Jacobi matrix. This partial differential equation for some function $\phi (p_2, p_3)$ reads as

4 $$\begin{aligned} -\dfrac{p_2}{p_3} \dfrac{\partial \phi }{\partial p_2} + \dfrac{\partial \phi }{\partial p_3} =0. \end{aligned}$$

A possible solution to Eq. (4) is $\phi = p_2p_3$. One option for reinstating this model’s identifiability is to reduce the number of parameters from 3 ($p_1, p_2, p_3$) to 2 ($p_1, \phi$). By introducing the scaled variable ${\tilde{x}}_2 \equiv x_2 / p_3$, we obtain the following reparameterised, identifiable model:

5 $$\begin{aligned} \frac{dx_{1}}{dt}&= p_1x_1^2+\phi x_1{\tilde{x}}_2, \qquad x_1(0) = x_{10} \ne 0, \end{aligned}$$

6 $$\begin{aligned} \frac{d{\tilde{x}}_{2}}{dt}&= x_1^2+x_1{\tilde{x}}_2, \qquad {\tilde{x}}_2(0) = 0. \end{aligned}$$

In view of the topic of this paper, we set out to find an alternative option for reinstating this model’s identifiability by investigating the role of the initial conditions. It is easy to confirm that the model’s unidentifiability can also be turned into identifiability simply by setting $x_2(0) \ne 0$. The Jacobi matrix computed for the scenario where $x_2(0) \ne 0$ is given in Eq. (7). Similar to Eq. (3), its 2 columns are related to system parameters $p_2$ and $p_3$ respectively. In contrast with the matrix in Eq. (3), the matrix in Eq. (7) has rank 2, so the linear dependence between its 2 columns is destroyed by setting $x_2(0) \ne 0$.

7 $$\begin{aligned} \frac{d {\varvec{G}}}{d \varvec{\theta ^{unid}}}(\varvec{x(0), \theta }) = \begin{pmatrix} 0 &{} 0 \\ x_1(0)x_2(0) &{} 0 \\ x_1(0)(p_3 x_1(0)^2+x_2(0)(x_1(0)+3p_1 x_1(0)+2p_2 x_5(0))) &{} p_2x_1(0)^2 \\ \end{pmatrix}. \end{aligned}$$

### Example 2

*Benchmark model with input signal* ($M_2$)

We now turn our attention to a benchmark model that has an input signal and was previously analysed by Saccomani et al.^4^. Here we analyse the local structural identifiability of the 4 unknown system parameters and so $\varvec{\theta }=[p_0,p_1,p_2,p_3]$. This example shows that problematic initial conditions are not necessarily zero values.

8 $$\begin{aligned} \frac{dx_{1}}{dt}&= -p_0u-p_2x_1-p_3x_2, \qquad x_1(0) = x_{10}, \end{aligned}$$

9 $$\begin{aligned} \frac{dx_{2}}{dt}&= p_3x_1x_2-p_1x_1, \qquad x_2(0) = \frac{p_1}{p_3}. \end{aligned}$$

**Figure 2:**
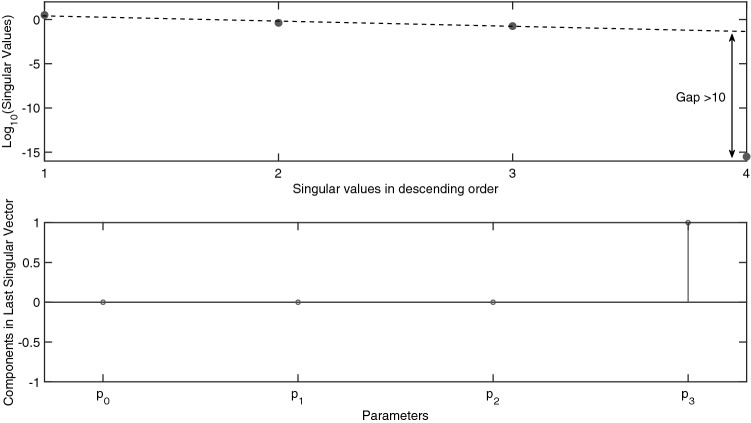
Identifiability signature of the benchmark model with input ($M_2$). (Top) Singular values: When state $x_1$ is measured and $x_2(0) = p_1/p_3$, the clear gap between the third and fourth singular values suggests rank deficiency of the sensitivity matrix. (Bottom) Entries in the last column of the right singular matrix: The nonzero entry corresponding to $p_3$ indicates that parameter $p_3$ is structurally unidentifiable.

Given the measured output $y=x_1$, Saccomani et al. show that when $x_2(0)=p_1/p_3$, $p_3$ is structurally unidentifiable. Our numerical results shown in Fig. 2, corroborate this result. Figure 2 shows entries of the last column of the ${\varvec{V}}$ matrix which related to the singular value beyond the gap. Its nonzero entry shows that parameter $p_3$ is not identifiable. To verify this numerical result symbolically, one begins by computing a set of Fliess series coefficients using Eq. (59) defined in the “Methods” section, ${\varvec{G}}(\varvec{x(0), \theta }) = \{ x_1(0), -p_2x_1(0)-p_3x_2(0), -p_0p_2, -p_0p_2^2+p_0p_3(-p_1+p_3x_2(0))\}$. Next, one substitutes the initial condition $x_2(0)=p_1/p_3$ into this series, and calculates partial derivatives of ${\varvec{G}}$ with respect to the unidentifiable parameter. For illustration, we compute the Jacobi matrix in this example with respect to all 4 system parameters. The columns are related to the parameters $p_0, p_1, p_2$, and $p_3$, respectively. Here we show the $5 \times 4$ matrix,

10 $$\begin{aligned} \frac{d {\varvec{G}}}{d \varvec{\theta }}(\varvec{x(0), \theta }) = \begin{pmatrix} 0 &{} 0 &{} 0 &{} 0 \\ 0 &{} -1 &{} -1 &{} 0 \\ -p_2 &{} 0 &{} -p_0 &{} 0 \\ -p_2^2 &{} 0 &{} -2p_0p_2 &{} 0 \\ -p_2^3 &{} 0 &{} -3p_0p_2^2 &{} 0 \\ \end{pmatrix}. \end{aligned}$$

The last column of Eq. (10) contains only zeros and accordingly system parameter $p_3$ is not structurally identifiable. The nontrivial null-space of the Jacobi matrix is ${\mathcal {N}} \left( \frac{d {\varvec{G}}}{d \varvec{\theta }}(\varvec{\theta }) \right) = \{0,0,0, 1 \}$. Our method confirms that this model’s identifiability is reinstated when $x_2(0) \ne {p_1}/{p_3}$. For example, if we would choose as initial condition $x_2(0) = {p_2}/{p_3}$, we would obtain the Jacobi matrix in Eq. (11), which has rank 4. Accordingly, we conclude that for this choice all 4 system parameters are locally identifiable.

11 $$\begin{aligned} \frac{d {\varvec{G}}}{d \varvec{\theta }}(\varvec{x(0), \theta }) = \begin{pmatrix} 0 &{} 0 &{} 0 &{} 0 \\ 0 &{} -2 &{} 0 &{} 0 \\ -p_2 &{} p_0 &{} 0 &{} 0 \\ -p_2^2 + (p_2-p_1)p_3 &{} -2p_0p_2 + p_0p_3 &{} -p_0p_3 &{} p_0(p_2-p_1) \\ \begin{aligned} p_2(p_2-p_1)p_3 + (p_2-p_1)p_3^2 \\ +p_2[-p_2^2 + (p_2-p_1)p_3] \end{aligned} &{} \begin{aligned} -p_0p_2^2 + p_0p_2p_3 \\ +2p_0(p_2-p_1)p_3 + p_0p_3^2 \\ +p_2(-2p_0p_2 + p_0p_3) \end{aligned} &{} -2p_0p_2p_3-p_0p_3^2 &{} \begin{aligned} 2p_0p_2(p_2-p_1) \\ +2p_0(p_2-p_1)p_3 \end{aligned} \\ \end{pmatrix}. \end{aligned}$$

### Example 3

*Erythropoietin (Epo) and Epo receptor (EpoR) interaction and trafficking* ($M_3$)

We now analyse a real-world systems biology model. In erythroid progenitor cells, which give rise to erythrocytes (commonly known as red blood cells), the dynamic properties of the Epo receptors determine how signals in the concentration of the ligand Epo are processed at the receptor level. This ultimately indicates how downstream signalling cascades such as the JAK2-STAT5 pathway are activated, which in turn leads to cellular responses such as differentiation and proliferation of erythrocytes^12^. The structural unidentifiability of this six state model which describes Erythropoietin (Epo) and Epo receptor (EpoR) interaction and trafficking was previously confirmed^12^. Given that four of the six initial conditions are zero, the aim here is to see whether changing these values from an experimental design perspective could address this model’s unidentifiability. The six model equations are^12,13^,

12 $$\begin{aligned}&\frac{d[Epo]}{dt} = -k_{on}[Epo][EpoR] + k_{on}k_D[Epo\_EpoR] + k_{ex}[Epo\_EpoR\_i], \end{aligned}$$

13 $$\begin{aligned}&\frac{d[EpoR]}{dt} = -k_{on}[Epo][EpoR] + k_{on}k_D[Epo\_EpoR] + k_t[EpoR(0)]-k_t[EpoR] + k_{ex}[Epo\_EpoR\_i], \end{aligned}$$

14 $$\begin{aligned}&\frac{d[Epo\_EpoR]}{dt} = k_{on}[Epo][EpoR] - k_{on}k_D[Epo\_EpoR] - k_e[Epo\_EpoR], \end{aligned}$$

15 $$\begin{aligned}&\frac{d[Epo\_EpoR\_i]}{dt} = k_e[Epo\_EpoR] - k_{ex}[Epo\_EpoR\_i] - k_{di}[Epo\_EpoR\_i] - k_{de}[Epo\_EpoR\_i], \end{aligned}$$

16 $$\begin{aligned}&\frac{d[dEpo\_i]}{dt} = k_{di}[Epo\_EpoR\_i], \end{aligned}$$

17 $$\begin{aligned}&\frac{d[dEpo\_e]}{dt} = k_{de}[Epo\_EpoR\_i]. \end{aligned}$$

The measured output defined by Raue et al.^12^ contains one additional unknown scaling parameter, *scale*:

18 $$\begin{aligned} y_1&= scale([Epo]+ [dEpo\_e]), \end{aligned}$$

19 $$\begin{aligned} y_2&= scale [Epo\_EpoR]. \end{aligned}$$

The initial values of [Epo] and [EpoR] are assumed to be unknown and so $\varvec{\theta }$ contains these and the 8 system parameters: $\varvec{\theta } = [k_{on},k_D, k_{ex},k_t,k_e,k_{di},k_{de}, scale, [Epo](0), [EpoR](0)]$. The initial conditions of all the remaining model states are zero:

20 $$\begin{aligned} \varvec{x(0)} = [[Epo](0), [EpoR](0), 0, 0, 0, 0]. \end{aligned}$$

The structural and practical identifiability of this model was assessed in a 2010 paper by calculating the profile likelihood related to each of its 10 unknown parameters^12^. Five structurally unidentifiable parameters were identified. Their identifiability was reinstated by assuming the value of [*Epo*](0) to be known^12^.

An alternative way to reinstate the model’s structural identifiability is the addition of one or more sensors to the model’s measured output . Which sensors might be added can efficiently be solved by determining a model’s minimal sensor set, the minimal set of sensors that needs to be measured to ensure model identifiability^14^. In this example, the identifiability can be reinstated by adding either state $[dEpo\_i]$ or $[dEpo\_e]$ to its measured output. In other words, measuring either ${\varvec{y}} = \{scale. ([Epo]+ [dEpo\_e]), scale . [Epo\_EpoR], \varvec{[dEpo\_i]}\}$ or ${\varvec{y}} = \{scale. ([Epo]+ [dEpo\_e]), scale . [Epo\_EpoR], \varvec{[dEpo\_e]}\}$ would ensure model identifiability. The physical limitations associated with this experimental implementation are not considered here.

**Figure 3:**
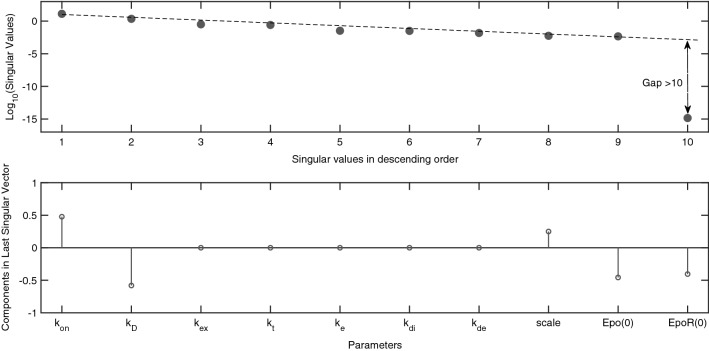
Identifiability signature of the Erythropoietin (Epo) and Epo receptor (EpoR) model ($M_3$). (Top) Singular values: When output ${\varvec{y}}=\{y_1,y_2\}$ is measured, and for the values defined in Eq. (20), with both [*Epo*](0),  [*EpoR*](0) nonzero. The clear gap between the spectrum of singular values suggests that the model is not structurally identifiable. (Bottom) Entries in the last column of the ${\varvec{V}}$ matrix related to the singular value beyond the gap: Nonzero entries indicate that parameters $k_{on}$, $k_D$, *scale*, [*Epo*](0) and [*EpoR*](0) are structurally unidentifiable and apparently totally correlated.

To understand the role this model’s initial conditions play in its unidentifiability, we analyse the model for the conditions stipulated in Eq. (20), with both [*Epo*](0),  [*EpoR*](0) assumed to be nonzero. The resulting identifiability signature is given in Fig. 3. The results indicate that $\varvec{\theta }^{unid}=\{k_{on}, k_D, scale, [Epo](0),$ $[EpoR](0)\}$ are unidentifiable. This result is symbolically verified by the base-vector spanning the nontrivial null-space: ${\mathcal {N}} \left( \frac{d {\varvec{G}}}{d \varvec{\theta }^{unid}}(\varvec{\theta }) \right) = \left\{ -\frac{k_{on}}{[EpoR](0)},\frac{k_D}{[EpoR](0)}, -\frac{scale}{[EpoR](0)},\frac{[Epo](0)}{[EpoR](0)}, 1 \right\}$.

We suspect that certain of the zero initial conditions reduce the dynamic information required to estimate accurate parameter values. To asses whether or not certain initial conditions contribute to the unidentifiability of the five parameters, we apply our numerical analysis of the model, each time selecting different combinations of nonzero initial values. The numerous iterations required in this analysis are made possible by the computational efficiency of the algorithm^9^. Assuming that the two unknown initial values, [*Epo*](0) and [*EpoR*](0) can also be zero, we find that the model’s identifiability can indeed be reinstated by setting certain initial conditions at nonzero values. The results in Table 1 reveal that there are three plausible experimental setups which would restore model identifiability. Scenarios 1 and 2 require that only a single initial condition holds a nonzero value. The first scenario is associated with the measured sensor in (19). In the third, a set of three specific nonzero initial conditions is defined. The Jacobi matrices for these three these scenarios can be computed similarly as done in Examples 1 and 2, given in Eqs. (7) and (11), respectively.

**Table 1:** List of plausible nonzero initial value combinations that ensure the structural identifiability of the Erythropoietin (Epo) and Epo receptor (EpoR) model ($M_3$).

Experimental scenario	Number of nonzero initial values	Nonzero initial value
1	1	$[Epo\_EpoR](0)$
2	1	$[Epo\_EpoR\_i](0)$
3	3	$[Epo](0), [EpoR](0), [Epo\_e](0)$

### Example 4

*JAK/STAT model* ($M_4$).

Here, we consider the well-known unidentifiable JAK/STAT model^15,16^. No literature has been published investigating the source of its unidentifiability and we address this question here by investigating the role of its initial conditions defined as^15^

21 $$\begin{aligned} {\varvec{x}}(0)=[x_1(0), \hdots , x_{14}(0)] =[1.3,x_2(0),0,0,0,2.8,0,165,0,0,0.34,0,0,0]. \end{aligned}$$

The 14 model equations are^15^

22 $$\begin{aligned} {\dot{x}}_1&= -\theta _1 u_1c_1x_1 - \theta _5 x_1 + \theta _6x_2, \end{aligned}$$

23 $$\begin{aligned} {\dot{x}}_2&= \theta _5x_1-\theta _6x_2, \end{aligned}$$

24 $$\begin{aligned} {\dot{x}}_3&= \theta _1 u_1c_1x_1 - \theta _2 x_3x_7, \end{aligned}$$

25 $$\begin{aligned} {\dot{x}}_4&= \theta _2x_3x_7-\theta _3x_4, \end{aligned}$$

26 $$\begin{aligned} {\dot{x}}_5&= \theta _3 x_4 - \theta _4 x_5, \end{aligned}$$

27 $$\begin{aligned} {\dot{x}}_6&= -\dfrac{\theta _7x_3x_6}{(1+\theta _{13}x_{13})}-\dfrac{\theta _7x_4x_6}{(1+\theta _{13}x_{13})}+\theta _8c_2x_7, \end{aligned}$$

28 $$\begin{aligned} {\dot{x}}_7&= \dfrac{\theta _7x_3x_6}{(1+\theta _{13}x_{13})}+\dfrac{\theta _7x_4x_6}{(1+\theta _{13}x_{13})}-\theta _8c_2x_7, \end{aligned}$$

29 $$\begin{aligned} {\dot{x}}_8&= -\theta _9x_8x_7+c_2\theta _{10}x_9, \end{aligned}$$

30 $$\begin{aligned} {\dot{x}}_9&= \theta _9x_8x_7-c_2\theta _{10}x_9, \end{aligned}$$

31 $$\begin{aligned} {\dot{x}}_{10}&= \theta _{11}x_9, \end{aligned}$$

32 $$\begin{aligned} {\dot{x}}_{11}&= -\theta _{12}c_1u_1 x_{11}, \end{aligned}$$

33 $$\begin{aligned} {\dot{x}}_{12}&= \theta _{12}c_1u_1 x_{11}, \end{aligned}$$

34 $$\begin{aligned} {\dot{x}}_{13}&= \dfrac{\theta _{14}x_{10}}{(\theta _{15}+x_{10})} - \theta _{16} x_{13}, \end{aligned}$$

35 $$\begin{aligned} {\dot{x}}_{14}&= \theta _{17}x_9. \end{aligned}$$

The defined output contains additional parameters $\theta _{18}, \hdots , \theta _{22}$

36 $$\begin{aligned} y_1&= x_1+x_3+x_4, \end{aligned}$$

37 $$\begin{aligned} y_2&= \theta _{18}(x_3+x_4+x_5+x_{12}), \end{aligned}$$

38 $$\begin{aligned} y_3&= \theta _{19}(x_4+x_5), \end{aligned}$$

39 $$\begin{aligned} y_4&= \theta _{20}x_7, \end{aligned}$$

40 $$\begin{aligned} y_5&= \theta _{21}x_{10}, \end{aligned}$$

41 $$\begin{aligned} y_6&= \theta _{22}x_{14}, \end{aligned}$$

42 $$\begin{aligned} y_7&= x_{13}, \end{aligned}$$

43 $$\begin{aligned} y_{8}&= x_{9}. \end{aligned}$$

**Figure 4:**
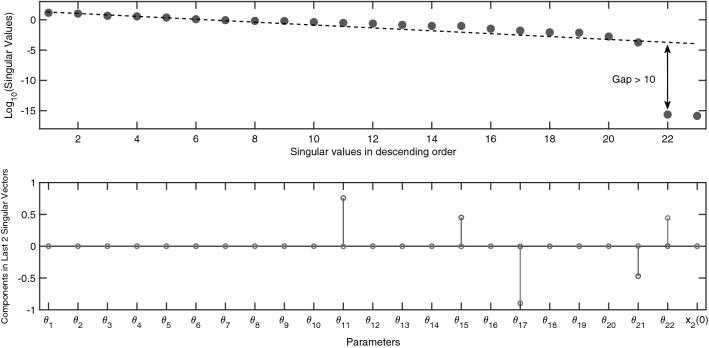
Identifiability signature of the JAK/STAT model ($M_4$). (Top) The 2 singular values beyond the gap calculated whilst measuring Eqs. (36)–(43) and using the initial conditions defined in Eq. (21). These suggest model unidentifiability and that there are two sets of totally correlated parameters. (Bottom) Entries in the last two columns of matrix ${\varvec{V}}$ related to the 2 zero singular values. The nonzero elements indicate the union between two potential sets of totally correlated parameters, $\{\theta _{11},\theta _{15}, \theta _{17}, \theta _{21}, \theta _{22}\}$.

With $x_2(0)$ assumed unknown, the identifiability of 23 unknown parameters in total must be analysed. The numerical results corroborate that this model is indeed unidentifiable for the initial conditions defined in Eq. (21), where $x_2(0) \ne 0$. This is apparent from the significant gap in the spectrum of singular values in Fig. 4. The two singular values beyond this gap suggest that the null-space contains two base-vectors and that there are two sets of totally correlated parameters. The unidentifiable parameters are the nonzero elements in the bottom of Fig. 4 and so the union of the elements in these sets is $\varvec{\theta }^{unid}=\{\theta _{11},\theta _{15},\theta _{17},\theta _{21},\theta _{22}\}$. The 2 base-vectors spanning the nontrivial null-space: ${\mathcal {N}} \left( \frac{d {\varvec{G}}}{d \varvec{\theta }^{unid}}(\varvec{\theta }) \right) =\left\{ 0,0,-\theta _{17}/ \theta _{22}, 0,1 \right\}$ and $\left\{ -\theta _{11}/ \theta _{21},-\theta _{15}/ \theta _{21},0,1,0\right\}$, give a clear indication as to which parameters are totally correlated. Using numerical results, the symbolic calculations associated with this example are significantly reduced since we only need to compute and analyse a Jacobi matrix with 5 columns instead of 23.

Systematically changing one or more of the zero values of the initial conditions in Eq. (21), we find that the identifiability is reinstated if we choose $x_{10}(0) \ne 0$ *and* $x_{14}(0) \ne 0$, leaving the other conditions at zero values. In hindsight, this conclusion can be understood from the output equations, $y_5=\theta _{21}x_{10}$ and $y_6=\theta _{22}x_{14}$, which contain the unidentifiable parameters $\theta _{21}$ and $\theta _{22}$, respectively. Taking $x_{10}(0) \ne 0$ *and* $x_{14}(0) \ne 0$ will ensure that sufficient dynamics is observed to enable the accurate estimation of all unknown parameters. We remark that setting $x_{10}(0) \ne 0$ destroys the base-vector $\left\{ 0,0,-\theta _{17}/ \theta _{22}, 0,1 \right\}$ and setting $x_{14}(0) \ne 0$ the base-vector $\left\{ -\theta _{11}/ \theta _{21},-\theta _{15}/ \theta _{21},0,1,0\right\}$.

### Example 5

*Pharmacokinetics model* ($$M_5$$).

Our final example contains only four model states and its global identifiability has been a topic of investigation since 2005. Unlike the previous four examples, this model is identifiable for the defined set of initial conditions. However, this does not imply that this holds for all initial conditions. Bellow we shall show that this model is an excellent example of the statement from Maria Saccomani, “It happens frequently in the global identifiability applications that the property holds only generically, i.e. except for a ‘thin’ set of initial conditions. In these situations the system is (incorrectly but forgivably) nevertheless declared to be (global) identifiable, excluding certain subsets of initial states”^4^.

We suspect that the model has sets of zero-valued problematic initial conditions that should be avoided during experimental design. To identify these sets, we perform an iterative search for zero-valued initial conditions which might render the model *unidentifiable*. This search is not exhaustive in the sense that we would search for all problematic combinations of initial values, as our aim is to find “a thin set of values” for which this model’s global identifiability as recorded in the literature, does not hold.

The model which comprises 4 model equations describes the ligands of the macrophage mannose receptor^17^

44 $$\begin{aligned} \frac{dx_{1}}{dt}&= \alpha _1(x_2-x_1) - \frac{k_av_mx_1}{(k_ck_a+k_cx_3+k_ax_1)}, \qquad x_1(0) = x_{10}, \end{aligned}$$

45 $$\begin{aligned} \frac{dx_{2}}{dt}&= \alpha _2(x_1-x_2), \qquad x_2(0) = 0, \end{aligned}$$

46 $$\begin{aligned} \frac{dx_{3}}{dt}&= \beta _1(x_4-x_3) - \frac{k_cv_mx_3}{(k_ck_a+k_cx_3+k_ax_1)}, \qquad x_3(0) = x_{30}, \end{aligned}$$

47 $$\begin{aligned} \frac{dx_{4}}{dt}&= \beta _2(x_3-x_4). \qquad x_4(0) = 0. \end{aligned}$$

The first state is measured, so the model’s output is defined as $y=x_1$. State $x_1$ represents the plasma enzyme concentration, $x_2$ its concentration in compartment 2, $x_3$ is the plasma concentration of the mannosylated polymer that acts as a competitor of glucose oxidase for the mannose receptor of macrophages, and $x_4$ is the concentration of this competitor in the extra vascular fluid of the organs accessible to this macro molecule^18^.

A 2005 publication^17^ on the topic of identifiability analysed this model using the differential algebra method. This method requires that functions ${\varvec{f}}$ and ${\varvec{h}}$, defined in the general descriptions Eqs. (49) and (51), be rational. This is clearly the case for the present model. The model was found to be globally identifiable. The analysis comprised two steps, where the unknown parameters were divided into two subsets $\{\alpha _1, \alpha _2, V_m, k_c \}$ and $\{ \beta _1, \beta _2, ka \}$, respectively. In this analysis the initial conditions were not taken into account. In a 2010 publication^19^ the model was reported to be globally identifiable, but only under the assumption that parameter $\alpha _2$ was known. No results could be obtained for the case with $\alpha _2$ unknown. The model was once more included in a 2011 publication^18^ which compared seven different identifiability analysis approaches. The local structural identifiability of six of the seven system parameters $\alpha _1,k_a,V_m,k_c,\beta _1$ and $\beta _2$, could be confirmed with the Taylor series method. In a recent publication^20^, the global identifiability result of Saccomani et al. was confirmed, this time also including the four initial conditions to the set of unknown parameters.

In our analysis we include $\alpha _2$ and all initial conditions as unknown parameters, so we take as vector of parameters to be estimated: $\varvec{\theta } = [\alpha _1,\alpha _2,k_a,V_m,k_c,\beta _1,\beta _2,x_1(0),x_2(0),x_3(0),x_4(0)]$. We proceed by performing an iterative search to identify problematic zero-valued initial conditions. Table 2 contains the initial value combinations for which we found the model to be not identifiable. These combinations may perhaps not be realistic in the context of this particular example. However, the emphasis here is to illustrate that the notion of global identifiability does not hold for this model. It also illustrates that the fast numerical method used in this paper allows for these kind of searches.

**Table 2:** Zero-valued initial condition combinations that result in the *loss* of structural identifiability of the Pharmacokinetics model ($M_5$).

Number of zero-valued initial conditions	States involved
2	$x_1(0) = x_2(0) = 0$
2	$x_3(0) = x_4(0) = 0$

**Figure 5:**
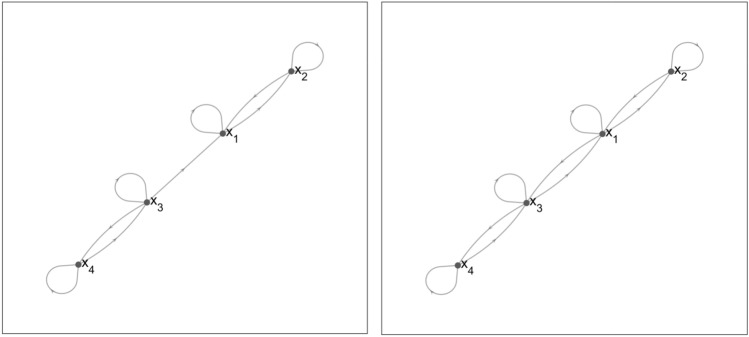
Directed graph of the Pharmacokinetics model ($M_5$). (Left) For the scenario $x_3(0)=x_4(0)=0$. The model is divided into 2 strongly connected components. (Right) For the scenario $x_3(0)\ne 0$ and $x_4(0)=0$. The additional connection between nodes $x_1$ and $x_3$ reduces the model down to a single component. The result is that a greater amount of information is transferred between the individual model states.

**Figure 6:**
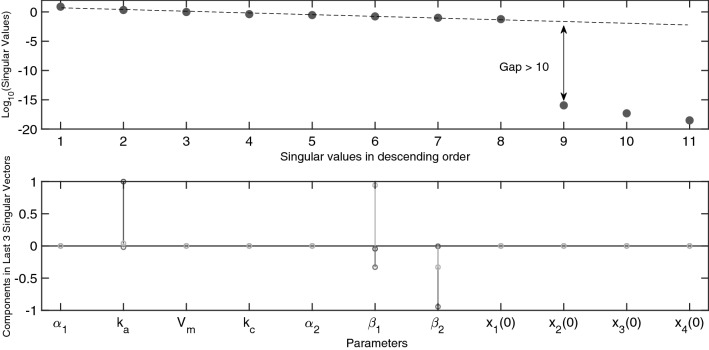
Identifiability signature of the pharmacokinetics model ($M_5$) for the experimental setup with $x_3(0)=x_4(0)=0$. (Top) The three singular values beyond the gap suggest that the sensitivity matrix is rank deficient and so the model is not identifiable. (Bottom) Entries in the last 3 columns of the ${\varvec{V}}$ matrix, related to the 3 zero valued singular values. The nonzero entries suggest that parameters $\{k_a, \beta _1, \beta _2\}$ are unidentifiable.

Figure 5 shows the change in the structure of the directed graph for the scenario where $x_3(0)=x_4(0)=0$ (left). For this case, the graph is divided into two strongly connected components with state $x_1$ and $x_2$ forming the first component and $x_3$ and $x_4$ the second. Notice that when $x_3(0) \ne 0$, that there is an additional connection between the nodes related to states $x_1$ and $x_3$ (right). This reduces the model structure down to one single component and therefore when $x_3(0) \ne 0$, information can flow from the measured state $x_1$ to the model equations pertaining to $x_3$ and $x_4$. As apparent from this directed graph, the identifiability signature in Fig. 6 shows that parameters $k_a, \beta _1$ and $\beta _2$, of which the latter two are exclusively related to the differential equations of states $x_3$ and $x_4$, are not identifiable. This result is corroborated by the symbolically computed $5 \times 7$ Jacobi matrix. in which each column is related to 1 of the 7 unknown system parameters, $\alpha _1, k_a,V_m,k_c, \alpha _2, \beta _1,\beta _2$. Since the columns related to parameters $k_a, \beta _1$ and $\beta _2$ contain only zero elements, we may immediately conclude that these parameters cannot be estimated. We symbolically calculated the base-vectors spanning the nontrivial null-space and found that ${\mathcal {N}} \left( \frac{d {\varvec{G}}}{d \varvec{\theta }}(\varvec{\theta }) \right) =\left\{ 0,1,0,0,0,0,0 \right\}$, $\left\{ 0,0,0,0,0,1,0 \right\}$, and $\left\{ 0,0,0,0,0,0,1 \right\}$. This tells us that there is no other totally correlated set of parameters. This is also in complete agreement with the identifiability signature in Fig. 6 which shows precisely 3 numerically zero-valued singular values. This result was obtained within 0.5 s on an Intel Core i7 processor with 8 GB RAM using the application described in^10^. This is comparable with EAR when the initial conditions are *not* parametrised. For parametrised initial conditions, differences in computation times were observed in favour of the sensitivity-based application from^10^.

$$\begin{aligned}& \frac{d {\varvec{G}}}{d {\boldsymbol{\theta} }}(\varvec{x(0)}, {\boldsymbol{\theta}} ) \\& \quad =\left(\begin{array}{lllllll} 0 & 0 & 0 & 0 & 0 & 0 & 0 \\ -x_1(0)+x_2(0) & 0 & \frac{-x_1(0)}{(k_c+x_1(0))} & \frac{V_m x_1(0)}{(k_c+x_1(0))^2} & 0 & 0 & 0 \\ \frac{((2 \alpha _1+\alpha _2)(k_c+x_1(0))^2(x_1(0)-x_2(0))+V_m(2 k_c x_1(0)+x_1(0)^2-k_c x_2(0)))}{(k_c+x_1(0))^2} & 0 & \hdots & \hdots & \alpha _1 (x_1(0)-x_2(0)) & 0 & 0 \\ \hdots & 0 & \hdots & \hdots & \hdots & 0 & 0 \\ \hdots & 0 & \hdots & \hdots & \hdots & 0 & 0 \end{array}\right). \end{aligned}$$

## Discussion

The results in this paper show the importance of including initial values in the identifiability analysis of any model. We showed how to identify problematic initial conditions that may result in loss of identifiability. The insight gained from this approach provides modellers with an extra tool to turn an unidentifiable model into a identifiable one, e.g., by avoiding initial values that may cause problems. This is especially useful during the design of the experiments. We also showed that thanks to the algorithm’s computational efficiency, one can detect these problematic values not only for small toy models but also for realistic system biology models that are usually fairly large.

Highlights include the identification of problematic initial conditions of the well-known JAK/STAT model comprising 14 model equations (Example 4) and the identification of problematic values that result in the loss of local identifiability of a Pharmacokinetics model, a model classified as globally identifiable in the past (Example 5).

Here, we mainly used our method to identify problematic sets of zero-valued initial conditions. The computational efficiency of the method allows for extensive searches in the space of initial conditions. However, nonzero initial conditions may also cause loss of identifiability and then the question that begs to be answered is “How does one go about identifying potential problematic sets of nonzero initial values?” This remains a challenging problem since in general the space of initial conditions is infinitely dimensional. The insight that the problematic sets form a thin subspace may be of help here. To find such manifolds one could think of first detecting one problematic point, after which the rest of the manifold could be traced via continuation. Another promising avenue to be pursued in the future is the investigation of systems in *steady state*. It is well-known that the estimation of certain parameters of such systems might be problematic, since the data do not contain enough informative dynamics for the accurate parameter estimation. Initial conditions that give rise to a steady state could thus rather be avoided. Given the size of modern systems biology models, any future research requires an algorithm that can quickly analyse identifiability. The approach followed in this paper provides such an algorithm.

As a final point, we wish to mention that caution should always be taken when analysing and giving judgement on a model’s structural identifiability. Given that this is a difficult property to analyse, for non-linear models in particular, no single method exists that can be applied to all models without fail. As an example, consider the following 1 state model:

48 $$\begin{aligned} \frac{dx_{1}}{dt}&= 1 + \theta _1x_1^2,\;\;\; x_1(0) = 0. \end{aligned}$$

For the defined output $y=x_1$, some of the methods mentioned in the introduction incorrectly classify parameter $\theta _1$ as unidentifiable, when in actual fact it is identifiable. By first applying our numerical step, one finds that the model is indeed identifiable and accordingly, no further analyses are required, eliminating the risk of obtaining incorrect results. This proves that using different methods in tandem will not only minimise computational times but will also improve computational correctness.

## Methods

### Model description

Ordinary differential equation models may be written in standard state-space form^21^:

49 $$\begin{aligned}&\dot{{\varvec{x}}}(t) = {\varvec{f}}_0({\varvec{x}}(t),\varvec{\theta }) + \sum _{i=1}^{k}{u}_i(t) {\varvec{f}}_i({\varvec{x}}(t),\varvec{\theta }), \end{aligned}$$

50 $$\begin{aligned}&{\varvec{x}}(0) = \varvec{x_0}, \end{aligned}$$

51 $$\begin{aligned}&{\varvec{y}}(t) = {\varvec{h}}({\varvec{x}}(t),\varvec{\theta }). \end{aligned}$$

State variables are contained in vector ${\varvec{x}}(t)$ with dimension *n*, system parameters in vector $\varvec{\theta }$ (dim$(\varvec{\theta })=p$) and the measured model outputs in vector ${\varvec{y}}(t)$ (dim$({\varvec{y}})=m$). The initial values of states may be unknown and in such instances, the initial condition vector may be parameterised through some additional parameters that then become part of the identification problem. The resulting unknown parameter vector then has dim($\varvec{\theta }$)$=p+n$^22^.

The state vector, ${\varvec{x}}$, evolves in time in $R^n$. Functions ${\varvec{f}}_i$, $i=0,\hdots ,k$, and ${\varvec{h}}$ are assumed to be analytical and $C^{\infty }$ functions, so that their partial derivatives of any order exist and are continuous^23^. Finally, a model’s input functions are contained in the vector ${\varvec{u}}(t)\equiv \{u_1,\hdots ,u_k\}$^22^.

### Local structural identifiability analysis

The identifiability detection method we propose combines both numerical and symbolic analyses. The methods starts with a numerical analysis of the rank of the so-called sensitivity matrix. This initial step pinpoints potentially unidentifiable parameters and initial conditions. In a second step a symbolic analysis is performed to check whether these parameters and initial conditions are indeed causing identifiability problems. In this discussion we deal with both steps separately. Note that at its core both the factorisation of the sensitivity matrix via SVD and the computation of the null-space of the Jacobi matrix have the same task: i.e. identifying linear dependencies between the partial derivatives of the output sensors with respect to the unknown parameters. The difference being that the sensitivity matrix represents the functions via values at different points whilst the matrix generated using Lie derivatives uses values of the derivatives at one point.

Since the second, symbolic step of our method usually involves only the analysis of a restricted number of parameters and initial conditions, the computational demand which is often found to be the curse of a symbolic analysis of the full model, is significantly reduced. The numerical results obtained from the first step can attractively be summarised in a so-called “identifiability signature”. This signature contains of a graphical presentation of the singular values resulting from a Singular Value Decomposition (SVD) of the sensitivity matrix. Structural unidentifiability is indicated by a *clear gap in the displayed singular values*^11^. Furthermore, the signature represents the components of the singular vectors corresponding with the close-to-zero singular values. The nonzero components reveal which parameters and initial conditions are expected to be problematic.

#### Numerical analysis

The numerical step of our method uses the sensitivity matrix function $\partial {\varvec{y}}/\partial \varvec{\theta }$ of the model output with respect to individual unknown model parameters. These sensitivities are calculated using the following 2 equations^9^:

52 $$\begin{aligned} \dfrac{d}{dt}\left( \dfrac{\partial {\varvec{x}}}{\partial \varvec{\theta }}\right)&= \dfrac{\partial {\varvec{f}}_0}{\partial {\varvec{x}}} \dfrac{\partial {\varvec{x}}}{\partial \varvec{\theta }} + \dfrac{\partial {\varvec{f}}_0}{\partial \varvec{\theta }} + \sum _{i=1}^{k} \left( \dfrac{\partial {\varvec{f}}_i}{\partial {\varvec{x}}} \dfrac{\partial {\varvec{x}}}{\partial \varvec{\theta }} + \dfrac{\partial {\varvec{f}}_i}{\partial \varvec{\theta }} \right) {u}_i, \end{aligned}$$

53 $$\begin{aligned} \dfrac{\partial {\varvec{y}}}{\partial \varvec{\theta }}&= \dfrac{\partial {\varvec{h}}}{\partial {\varvec{x}}}\dfrac{\partial {\varvec{x}}}{\partial \varvec{\theta }} + \dfrac{\partial {\varvec{h}}}{\partial \varvec{\theta }}. \end{aligned}$$

One obtains $\partial {\varvec{y}}/\partial \varvec{\theta }$ as a function of time by simultaneously integrating Eqs. (49) and (52) and substituting the solution into Eq. (53)^24^. By evaluating these sensitivities at discrete time points on an interval $\left[ t_0, \hdots , t_N \right]$ one constructs a sensitivity matrix, ${\varvec{S}}$. Matrix ${\varvec{S}}$ has $p+n$ columns when all initial values of model states are also unknown, with each column related to a specific parameter or initial condition, $\theta _i, i=1,\hdots ,p+n$. The sensitivity matrix thus reads as:

54 $$\begin{aligned} {\varvec{S}}(t_0,\hdots ,t_N,\varvec{\theta }) = \begin{pmatrix} \dfrac{\partial y_1}{\partial \theta _1}(t_0) &{} \ldots &{} \dfrac{\partial y_1}{\partial \theta _{p+n}}(t_0) \\ \vdots &{} \ddots &{} \vdots \\ \dfrac{\partial y_m}{\partial \theta _1}(t_0) &{} \ldots &{} \dfrac{\partial y_m}{\partial \theta _{p+n}}(t_0) \\ \vdots &{} &{} \vdots \\ \dfrac{\partial y_1}{\partial \theta _1}(t_N) &{} \ldots &{} \dfrac{\partial y_1}{\partial \theta _{p+n}}(t_N) \\ \vdots &{} \ddots &{} \vdots \\ \dfrac{\partial y_m}{\partial \theta _1}(t_N) &{} \ldots &{} \dfrac{\partial y_m}{\partial \theta _{p+n}}(t_N) \end{pmatrix}. \end{aligned}$$

A full ranked matrix ${\varvec{S}}$ is a sufficient condition for local structural identifiability^25,26^. The rank deficiency of the sensitivity matrix can be attributed to two factors: (1) an output may be insensitive to a specific parameter and so all entries in the matrix column pertaining to this parameter are zero. Accordingly the parameter is not identifiable. This phenomenon is observed in Examples 2 and 5 in the “Results” section. (2) Alternatively, a model output may be sensitive to a particular parameter, but this sensitivity is related to the sensitivity of the output to one or more other parameters^24^. The result is that certain columns of the sensitivity matrix are linearly dependent and so the parameters are totally correlated and not identifiable^27^. This can be seen in Examples 2, 3 and 4 in the “Results” section. We determine the numerical rank of the sensitivity matrix by applying a Singular Value Decomposition (SVD), in which the matrix ${\varvec{S}}$ is written as a sum of equally sized matrices that decrease in dominance^11^:

55 $$\begin{aligned} {\varvec{S}}(t_0,\hdots ,t_N,\varvec{\theta }) = u_1\sigma _1v_1^T+\hdots +u_{p+n}\sigma _{p+n}v_{p+n}^T. \end{aligned}$$

If all parameters and initial conditions are involved, there are $p+n$ singular values $\sigma _i$, arranged in descending order. The rank of ${\varvec{S}}$ is given by the number of nonzero singular values. Therefore zero-valued singular values indicate the rank-deficiency of ${\varvec{S}}$^28^. Due to numerical errors, singular values are seldom exactly zero and accordingly the following practical definition is used: zero-valued singular values are values that fall beyond a *distinct gap* in the spectrum of singular values^29^. It is up to the user to define a reliable width for the gap. In our examples, we take 10 decades on the logarithmic scale as reliable gap width. The presence of close-to-zero singular values, which are located beyond such a gap, indicate that the model may be unidentifiable. The parameters and initial values that may be involved follow from the nonzero entries in the columns, $v_i$, of the right singular matrix, that correspond with the close-to-zero singular values. The singular values and the unidentifiable parameters are graphically illustrated in an identifiability signature^11^. We show the signatures of each of the models in the 5 Examples in the Results section (cf. Fig. 1).

#### Symbolic calculations

The second step in our procedure is to verify the numerical results symbolically. This entails the symbolic calculation of the Jacobi matrix of a model. The computational demand often associated with computing this matrix is greatly reduced by using the preceding numerical outcomes^9^. Available software packages that can be used include amongst others: COMBOS^30^ and GenSSI2.0^31^. We use Lie derivatives and accordingly, only compute derivatives of the Lie derivatives with respect to the parameters that are suggested to be unidentifiable from the numerical analysis. We indicate them by $\varvec{\theta ^{unid}}$. We use the rank condition for local structural identifiability presented by Tunali and Tarn^32^.

The Jacobi matrix of a model with no control inputs ($u_1,u_2,\hdots ,u_k=0$) can be computed using Lie derivatives, where a Lie derivative is the directional derivative of the smooth function, ${\varvec{h}}$, in the direction of the drift vector field, ${\varvec{f}}_0$^24^. Mathematically it is defined as

56 $$\begin{aligned} {\mathcal {L}}_{{\varvec{f}}_0} {\varvec{h}}= \dfrac{\partial {\varvec{h}}}{\partial {\varvec{x}}} {\varvec{f}}_0. \end{aligned}$$

Successive Lie derivatives are calculated as

57 $$\begin{aligned} {\mathcal {L}}^i_{{\varvec{f}}_0} {\varvec{h}}= \dfrac{\partial {\mathcal {L}}^{i-1}_{{\varvec{f}}_0} {\varvec{h}}}{\partial {\varvec{x}}} {\varvec{f}}_0. \end{aligned}$$

The symbolic algebra package Kwatny’s ProPac add-on for Mathematica can be used to calculate these Lie derivatives^22^. In a generating series expansion, successive Lie derivatives of the output vector function ${\varvec{h}}$ are calculated. Parameterising the unknown initial conditions and so regarding them as additional parameters, the Jacobi matrix may have up to $p+n$ columns, each related to an individual parameter. Finally, the Jacobi matrix is computed by calculating partial derivatives of the generating series coefficients with respect to the unknown parameters. The symbolic matrix associated with the analysis of all system parameters and initial conditions reads as^9^,

58 $$\begin{aligned} \frac{\partial {\varvec{G}}}{\partial \varvec{\theta }}(\varvec{\theta }) = \begin{pmatrix} \dfrac{\partial {\varvec{h}}}{\partial \theta _1} &{} \ldots &{} \dfrac{\partial {\varvec{h}}}{\partial \theta _{p+n}} \\ \dfrac{\partial {\mathcal {L}}_{{\varvec{f}}_0}{\varvec{h}}}{\partial \theta _1} &{} \ldots &{} \dfrac{\partial {\mathcal {L}}_{{\varvec{f}}_0}{\varvec{h}}}{\partial \theta _{p+n}} \\ \dfrac{\partial {\mathcal {L}}^2_{{\varvec{f}}_0}{\varvec{h}}}{\partial \theta _1} &{} \ldots &{} \dfrac{\partial {\mathcal {L}}^2_{{\varvec{f}}_0}{\varvec{h}}}{\partial \theta _{p+n}} \\ \vdots &{} \ldots &{} \vdots \end{pmatrix}. \end{aligned}$$

A sufficient condition for structural identifiability is that $\frac{\partial {\varvec{G}}}{\partial \varvec{\theta }}(\varvec{\theta })$, defined in Eq. (58), has full rank $p+n$^9^. A lower rank is equivalent to it having a nontrivial null-space^33^. The elements in such a nontrivial null-space reveal which parameters and initial conditions are involved in one or more correlated sets.

When evaluating models of the form defined in Eqs. (49)–(51) with $u_1,u_2,\hdots ,u_k\ne 0$, individual input functions must be incorporated into the symbolic calculations^21,34^. An output is now expanded in a so-called Fliess series^32^ with respect to time and *input signals*. The coefficients of such a series are ${\varvec{h}}({\varvec{x}}(0),\varvec{\theta })$ and

59 $$\begin{aligned} {\mathcal {L}}_{\varvec{f_{j_0}}} \hdots {\mathcal {L}}_{\varvec{f_{j_q}}} {\varvec{h}}({\varvec{x}}(t),\varvec{\theta })|_0. \end{aligned}$$

The notation $\varvec{f_{j_0}}, \hdots ,\varvec{f_{j_q}}$ represents all possible combinations of the vector fields $\{ \varvec{f_{j}}, j=0,\hdots ,k\}$^9,21^. Furthermore, $|_0$ specifies that this Jacobi matrix is evaluated in the point ${\varvec{x}}(0)$. It is clear that this procedure leads to a fast expanding number of terms if the values of *k* (the number of input signals) and *p* (the number of system parameters) increase. To give an impression of the Jacobi matrix associated with the full model in Eq. (52) for $k=1$, we restrict the number of parameters to 1, e.g. $\theta _1$, and the number of initial values also to 1, e.g. $x_0$. In that very basic case, the Jacobi matrix reads as^9^:

60 $$\begin{aligned} \frac{\partial {\varvec{G}}}{\partial \varvec{\theta }}(\varvec{\theta }) = \begin{pmatrix} \dfrac{\partial {\varvec{h}}}{\partial \theta _1} &{} \dfrac{\partial {\varvec{h}}}{\partial x_1(0)} \\ \dfrac{\partial {\mathcal {L}}_{{\varvec{f}}_0}{\varvec{h}}}{\partial \theta _1} &{} \dfrac{\partial {\mathcal {L}}_{{\varvec{f}}_0}{\varvec{h}}}{\partial x_1(0)}\\ \dfrac{\partial {\mathcal {L}}_{{\varvec{f}}_1}{\varvec{h}}}{\partial \theta _1} &{} \dfrac{\partial {\mathcal {L}}_{{\varvec{f}}_1}{\varvec{h}}}{\partial x_1(0)}\\ \dfrac{\partial {\mathcal {L}}_{{\varvec{f}}_1} {\mathcal {L}}_{{\varvec{f}}_0}{\varvec{h}}}{\partial \theta _1} &{} \dfrac{\partial {\mathcal {L}}_{{\varvec{f}}_1}{\mathcal {L}}_{{\varvec{f}}_0}{\varvec{h}}}{\partial x_1(0)}\\ \dfrac{\partial {\mathcal {L}}_{{\varvec{f}}_0} {\mathcal {L}}_{{\varvec{f}}_1}{\varvec{h}}}{\partial \theta _1} &{} \dfrac{\partial {\mathcal {L}}_{{\varvec{f}}_0}{\mathcal {L}}_{{\varvec{f}}_1}{\varvec{h}}}{\partial x_1(0)}\\ \dfrac{\partial {\mathcal {L}}_{{\varvec{f}}_1} {\mathcal {L}}_{{\varvec{f}}_1} {\mathcal {L}}_{{\varvec{f}}_0}{\varvec{h}}}{\partial \theta _1} &{} \dfrac{\partial {\mathcal {L}}_{{\varvec{f}}_1} {\mathcal {L}}_{{\varvec{f}}_1}{\mathcal {L}}_{{\varvec{f}}_0}{\varvec{h}}}{\partial x_1(0)}\\ \dfrac{\partial {\mathcal {L}}_{{\varvec{f}}_1} {\mathcal {L}}_{{\varvec{f}}_0} {\mathcal {L}}_{{\varvec{f}}_0}{\varvec{h}}}{\partial \theta _1} &{} \dfrac{\partial {\mathcal {L}}_{{\varvec{f}}_1} {\mathcal {L}}_{{\varvec{f}}_0}{\mathcal {L}}_{{\varvec{f}}_0}{\varvec{h}}}{\partial x_1(0)}\\ \vdots &{} \vdots \\ \dfrac{\partial {\mathcal {L}}_{{\varvec{f}}_{j_0}} \hdots {\mathcal {L}}_{{\varvec{f}}_{j_q}} {\varvec{h}}}{\partial \theta _1} &{} \dfrac{\partial {\mathcal {L}}_{{\varvec{f}}_{j_0}} \hdots {\mathcal {L}}_{{\varvec{f}}_{j_q}} {\varvec{h}}}{\partial x_1(0)}\\ \vdots &{} \vdots \end{pmatrix}, \end{aligned}$$

where $j_0, j_1, \hdots , j_q \in [0,1]$. Usually, a null-space will emerge if this matrix has only few rows. However, in the process of adding additional rows, two things may happen: either the basis vectors of this null-space are destroyed at some stage, indicating that the system is identifiable, or this null-space will persist regardless of the number of rows added, indicating that the system is structurally unidentifiable.

## Data Availability

MATLAB code is available at https://sourceforge.net/projects/structural-identifiability/files/ with the user application available upon request from the author hans.stigter@wur.nl.
